# Automated Algorithm for Removing Clutter Objects in MMS Point Cloud for 3D Road Mapping

**DOI:** 10.3390/s20154076

**Published:** 2020-07-22

**Authors:** Jisang Lee, Suhong Yoo, Seunghwan Hong, Mohammad Gholami Farkoushi, Junsu Bae, Ilsuk Park, Hong-Gyoo Sohn

**Affiliations:** 1 School of Civil and Environmental Engineering, Yonsei University, 50 Yonsei-ro, Seodaemun-gu, Seoul 03722, Korea; ontheground@yonsei.ac.kr (J.L.); swennoir@yonsei.ac.kr (S.Y.); mohamad_gholami@yonsei.ac.kr (M.G.F.); junsu510@yonsei.ac.kr (J.B.); 2 Stryx co., 50-1 Yonsei-ro, Seodaemun-gu, Seoul 03722, Korea; cto@stryx.co.kr (S.H.); ceo@stryx.co.kr (I.P.)

**Keywords:** mobile mapping system, instance segmentation, point cloud removal, HD map, clutter objects

## Abstract

Road information high definition maps (HD map) contain information about the facilities around the roads and are often constructed through a mobile mapping system (MMS). Although constructing an HD map is essential for road maintenance and the application of autonomous driving in the future, it is problematic to acquire the data of objects other than the facilities in an unstructured form while operating the MMS. In this study, the researchers define this object data as clutter objects and present a method of automatic removal using characteristics of the MMS and image segmentation techniques. By applying the method to 10 KITTI (Karlsruhe Institute of Technology and Toyota Technological Institute at Chicago) datasets, clutter objects were removed with an average overall accuracy of 91% with 0% (0.448%) error of commission for the complete point cloud map.

## 1. Introduction

High definition point cloud maps (HD map), constructed using mobile mapping systems (MMS), contain information about the static environment around roads [1,2,3,4], which is essential for road maintenance and future application of autonomous driving [5,6,7]. The construction of HD map using MMS is achieved by using the following procedure and characteristics. First, an MMS vehicle mounted with several fused sensors such as camera, LiDAR, and GNSS/INS moves on a road that is always in operation. Second, data are acquired for each data acquisition frequency and fused at the frame level. Third, by accumulating the fused frame data in temporal order, an HD map is constructed with red, green, blue (RGB), an location data [1].

**Figure 1:**
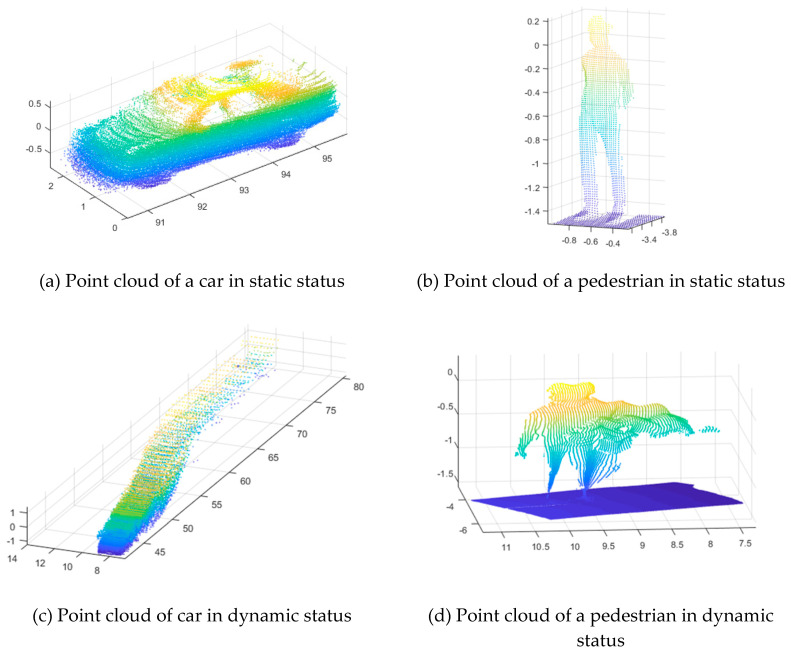
Examples of the distorted shape of clutter objects after data acquisition.

However, during the map creation process, the MMS vehicle being operated on an active road may create a problem. Although the main aim of an HD map is to display the road facility information, data of clutter objects, such as cars and pedestrians, are inevitably acquired. Removing these data is an important task while editing the HD map for road facility information [8]. However, removing clutter objects is not that easy because of a typical problem that is encountered due to the data acquisition method of the MMS. When LiDAR, which is the core technology equipment used in the MMS point cloud construction, scans the surrounding environment, the laser emitted from the device sweeps along the side of the target objects. Moreover, because the clutter objects, such as cars on the road, often move with the MMS vehicle during the data scanning, the acquired data show a completely different shape from the data acquired in a static state. Figure 1 shows the distorted shape of clutter objects in dynamic status.

Such distorted object datasets are generated over the entire road length, and the existing automatic point cloud detection methods that are applicable to static object data cannot detect them [9,10,11]. Therefore, manual removal is the best approach to remove the point cloud data of these objects in dynamic state, but it is labor-intensive. 

Therefore, this study suggests a method to automatically remove the clutter objects on the road by considering them as clutter objects by using image segmentation and the spatial relationship between the devices that make up the MMS. The terminology “clutter objects” is defined as objects that frequently appear and acquired unintendedly in constructing road facility map on roads, such as pedestrians, cars, trucks, bicycles, trains, motorcycles, and buses. Therefore, for the purpose of this research, “clutter objects” is defined as the point cloud data of the unintended objects.

## 2. Materials and Methods

### 2.1. Data Acquisition

#### 2.1.1. Basic MMS Information

An MMS generally acquires data simultaneously while the moving platform on which it is mounted, such as a car or bicycle carrying an integrated sensor composed of GNSS/INS, Camera, and LiDAR, moves in the acquisition area [1]. The GNSS/INS device, which comprises the integrated sensor, acquires the absolute object position and posture information to calculate the heading. The camera acquires the images of the surroundings for identification of the objects, and LiDAR acquires the depth information of the surroundings to capture the object position accurately. And the RGB data acquired from the camera can be given to the point cloud to facilitate object identification. The KITTI (Karlsruhe Institute of Technology and the Toyota Technological Institute at Chicago) dataset was used in this study as the representative open MMS dataset, and the specifications can be accessed on the KITTI website [12]. Each sensor was assembled rigidly on the basis of the GNSS/INS specifications, and the heading and position of each sensor was accurately calculated in real time by using the boresight and lever-arm parameters of the sensors.

**Figure 2:**
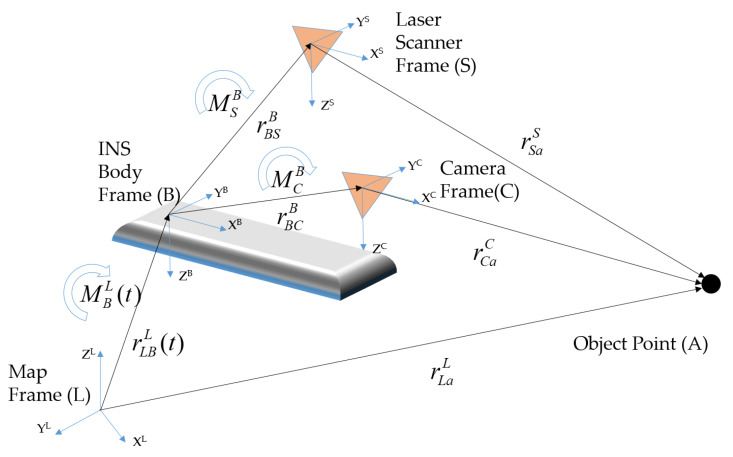
Spatial configuration of mobile mapping system (MMS) component devices.

All the sensors in the MMS were time-synchronized, so that they were configured to acquire data almost simultaneously with the moving MMS. These simultaneous data acquisitions occurred within a short frequency period of 10 Hz, and formed a set to record the spatial data concretely. A set of data from the GNSS/INS, camera image, and LiDAR point cloud acquired at the same moment can be integrated within the same frame, because these data are also spatially synchronized based on the previously mentioned boresight and lever-arm parameters, as shown in Figure 2 [1,13,14].

As shown in the figure, the coordinate relationship among the object point (A), laser scanner frame (S), camera frame (C), INS body frame (B), and map frame (L) can be expressed mathematically through rotation and translation among the coordinate systems. The coordinate system of the image and the laser scanner is integrated based on the INS coordinate system, which is then projected onto the map coordinate system. The mathematical model expressing the geometric relationship can be defined by Equation (1).

(1) $$r_{La}^{L}=r_{LS}^{L}\left(t\right)+M_{B}^{L}\left(t\right)\left(M_{S}^{B}r_{Sa}^{S}+r_{BS}^{B}\right),$$

where $r_{\mathrm{La}}^{L}$ represents the coordinate of *A* in the map frame, *t* the observation time, $r_{LS}^{L}(t)$ the position of the INS body frame in the map frame, $M_{B}^{L}\left(t\right)$ the rotation matrix from the INS body frame to the map frame, $M_{S}^{B}$ the rotation matrix from the laser scanner frame to the INS body frame, $r_{\mathrm{BS}}^{B}$ the position of the laser scanner in the INS body frame, and $r_{\mathrm{Sa}}^{S}$ the position of object point in the laser scanner frame.

**Figure 3:**
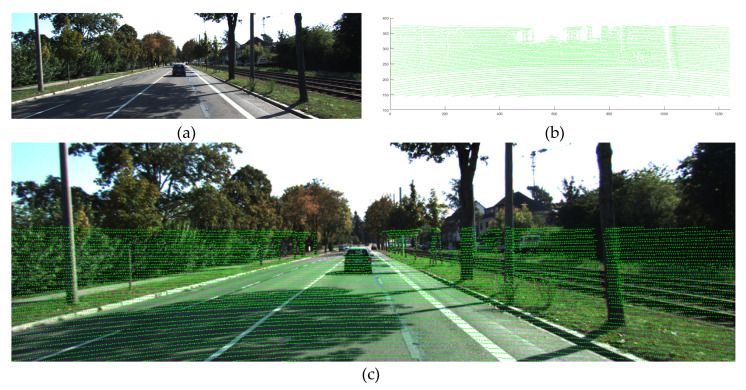
(**a**) Acquired image; (**b**) Acquired point cloud; (**c**) Point projection onto image through spatial registration.

Each individually acquired data value is integrated and expressed as shown in Figure 3. By projecting the point cloud with absolute coordinates onto the images, the absolute coordinates of the desired objects can be obtained. This set is a frame of acquired spatial information, and such sets of spatial information are continuously acquired in the 10 Hz frequency.

#### 2.1.2. KITTI Dataset

The KITTI dataset has been obtained from “Karlsruhe Institute of Technology and the Toyota Technological Institute at Chicago” and constructed by using MMS in the Karlsruhe region and on the surrounding highways. The dataset and the MMS parameters are open to public on the KITTI website [12,15]. The KITTI dataset consists of 28 urban areas, 16 residential areas, 12 roads, 10 campuses, and 80 human figures. The researchers selected 10 datasets that included sufficient road information to suit the nature of this experiment, as shown in Table 1.

**Figure 4:**
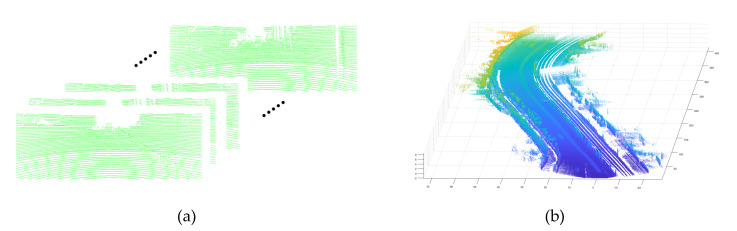
(**a**) Point cloud frames and (**b**) Point cloud map.

The KITTI data are provided framewise as general MMS data sets are. And when the frames of data are accumulated according to the time sequence and the trajectory acquired by the GNSS/INS, they compose an overall point cloud map as shown in Figure 4.

### 2.2. Methodology

#### 2.2.1. Schematic Workflow

**Figure 5:**
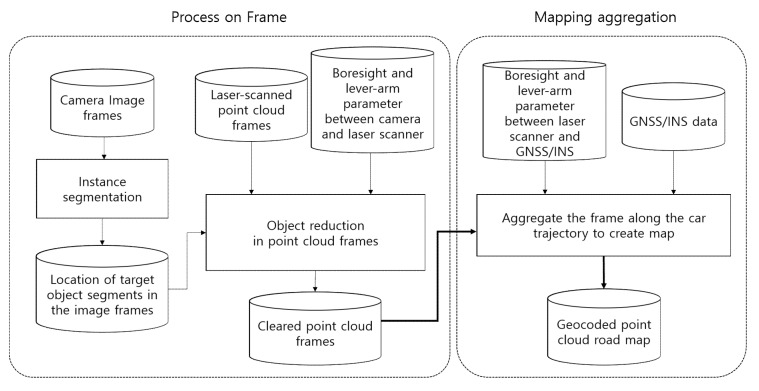
Schematic workflow.

To remove the noisy point clouds, the researchers used a typical characteristic of the MMS data acquisition. As mentioned earlier, MMS data are first acquired as frames by individual devices such as the GNSS/INS, camera, and LiDAR, and then synchronized temporally and spatially. Thereafter, the point cloud map is generated by accumulating these frames. Removing clutter point clouds is not an easy task, as they tend to be acquired in a distorted shape, and the existing detection methods are based on recognizing the point cloud data in the original object shape. Therefore, a clean point cloud map can be obtained by removing the clutter objects at the frame level and then accumulating the clean point cloud frames. As the image acquired with the camera and the point cloud acquired with the LiDAR are synchronized, the location of the target clutter objects in the image and point cloud can be identified and removed at the frame level itself. The schematic workflow is shown in Figure 5.

#### 2.2.2. Mask R-CNN for Instance Segmentation

To locate and remove the clutter objects, Mask R-CNN, a technique for instance segmentation is applied to the RGB images acquired from the camera. Mask R-CNN combines semantic segmentation and object detection to produce an effect of instance segmentation [16]. Based on Mask R-CNN paper published by Facebook AI Research, the code was written in Python 3 and Keras by Matterport and adapted to suit our purpose [17]. The Matterport code was edited for making it appropriate to handle the KITTI dataset. As the clutter objects to be removed from the road are limited to common objects which can be encountered in common road situation, we used weights pre-trained with Microsoft Common Objects in Context dataset (MS-COCO dataset), a dataset with 330K images with 200K labels of these common objects, which includes on road objects such as cars, pedestrians, buses, bikes, motorbikes, cans, and trains [18]. To apply the algorithm to the KITTI data, several changes were made to the Matterport code. First, to enable batch processing of data for various image sizes, the original paper mentioned that the image size should be resized to 1000 square pixels. However, as the images provided by the KITTI dataset were in 1242 × 375 dimensions, zero padding was performed on the insufficient side (375 pixels) to scale it up to 1242 and then the 1242 square pixel images were resized to 1000 square pixels. Second, a smaller value of learning rate was used than 0.02, which was used in the original paper. Because the authors of the original research used gradient descent methods from the open libraries, which are different from ours, the learning rates were modified. Mask R-CNN provides an instance segmentation result in pixels. 

#### 2.2.3. Extra Pixel Padding

**Figure 6:**
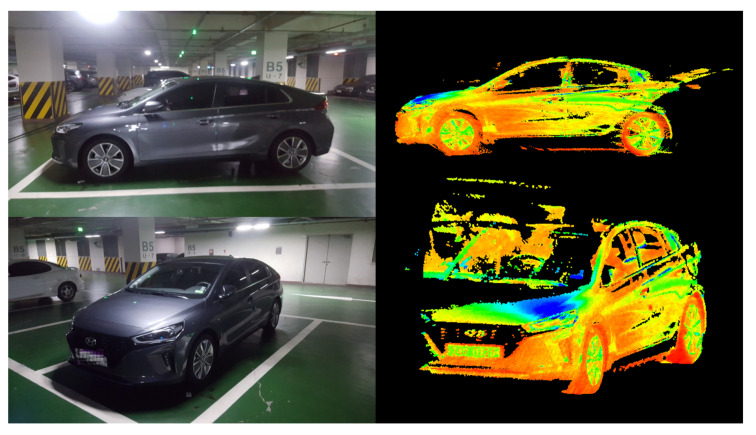
Examples of extended noise points reflected by the car surface.

In the case of vehicles such as cars and trucks, which were the main clutter source in this research, the external coating and glass part of the vehicle body tends to reflect or transmit the laser emitted from LiDAR so that frequently a different shape is acquired from its original one. Especially, as shown in Figure 6, a larger shape is acquired because of the laser bounce or transmission from the vehicle surface. Therefore, to accurately detect and remove such shapes, the original result mask from Mask R-CNN was extended by 30 pixels. The incremented number for the pixel expansions was chosen for the best results through repetitive experiments.

## 3. Results

### 3.1. Results of Clutter Object Removing

**Figure 7:**
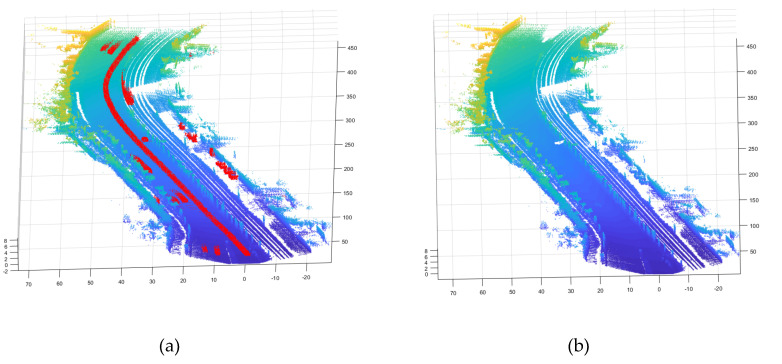
(**a**) Point cloud map with clutter objects, (**b**) Cleared point cloud map.

Looking at dataset No. 56 (2011_09_26_drive_0056_sync) as shown in Figure 7, the figure on the left shows the point cloud data created as a map, and the point clouds shown in red represent clutter objects to be removed, which have been manually selected for reference. The figure on the right shows the cleared point cloud map after applying the proposed method, and the red points were effectively removed. The results for the remaining nine datasets can be found in the appendix.

### 3.2. Accuracy Assessment

**Figure 8:**
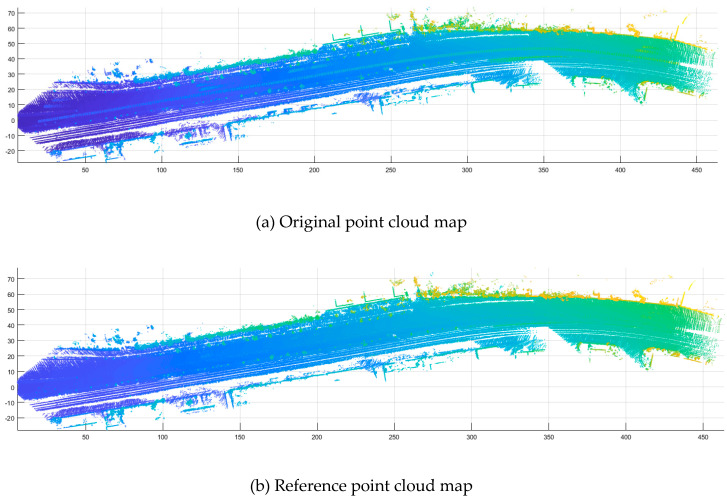

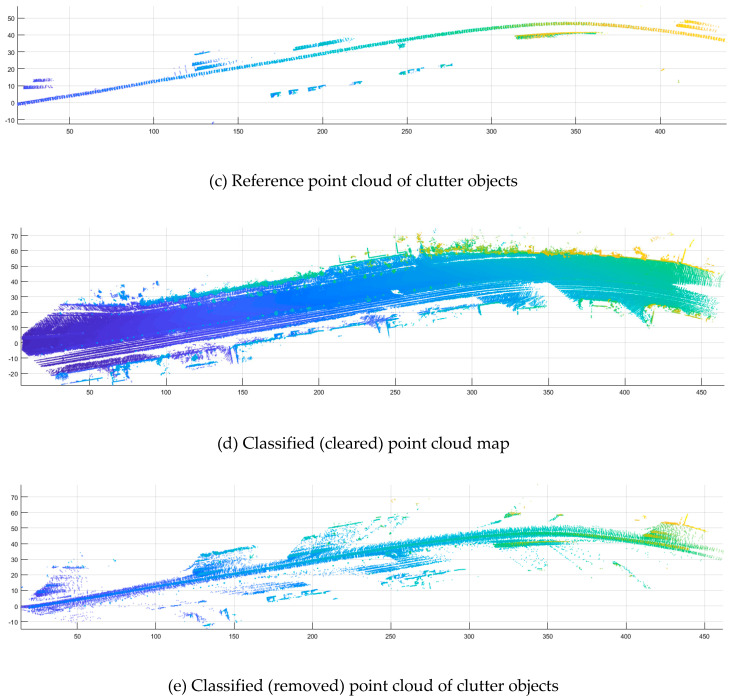
Results of clutter object removal.

Accuracy assessment was performed using overall accuracy, and error of omission and commission indicators. Figure 8 shows the original point cloud map, the reference point cloud map, reference point cloud of clutter objects, classified (cleared) point cloud map, and classified (removed) point cloud of clutter objects, respectively. This is a representative visualization of dataset No. 56.

The resulting error matrix of the data is shown in Table 2. Error of omission is the probability that some clutter objects will be missed during the classification. Error of commission is the probability that a classification failure will occur. Overall accuracy means the accuracy of the overall classification.

These parameters can be expressed by a formula as shown in Table 3**.** As shown in Table 2 and Table 3, the overall accuracy was 95%, error of omission for clutter objects was 21%, error of commission for clutter objects was 73%, error of omission for map was 8%, and the error of commission for map was 1%. The accuracy assessment for the remaining data can be found in the appendix.

## 4. Discussion

Using the proposed method, the researchers were able to automatically remove clutter objects from the road information point cloud map. Until now, the only way to remove the clutter objects was to install a point cloud editing program and manually identify the clutter objects to remove it directly; however, using the proposed automatic reduction method, the researchers experienced increased convenience in constructing the point cloud map. 

The limitation of this algorithm is that the overall accuracy is dependent on the accuracy of the image segmentation result. As the clutter object identification procedure starts with a visual identification of clutter objects on the image acquired with the camera, the accuracy of the point cloud result will not be high enough if the result of image segmentation is not sufficiently accurate. This problem is expected to be gradually solved through the development of more efficient image segmentation methods. In addition, the study is conducted under the assumption that frame-by-frame raw data can be obtained, since all point cloud data acquired by MMS is acquired as raw data in frame units. Therefore, in the future, study should be conducted on removing clutter objects from the as-constructed point cloud map after post-processing.

Moreover, looking at the evaluation results, the error of commission for clutter objects is quite high, which is caused by pixel expansion. As explained earlier, in the case of the car point cloud, which is the main clutter object type prevalent in the road information, the acquired object shapes are more exaggerated than the original ones due to the reflection of the laser point by its external coating. Therefore, to catch all the noise accurately, a 30-pixel buffer was applied, which caused the noise to be exaggerated than the actual size, resulting in a relatively high error of commission for clutter objects. However, this is not a big problem for using the result map, because the true map points in the area wrongly identified as clutter points due to the exaggerated buffer in a frame can be filled in the next frame when the clutter object moves away from that area. As evidence, the integrity of the map construction is confirmed by the low error of commission of 1% for the final map.

In this study, the researchers have proposed a method to remove clutter object data from a road information point cloud map. The clutter objects in the road information point cloud map refers to an object that is moving, and thus does not belong to the road facilities. In this research, only general objects were used as identifiable clutter objects and removed, and the MS-COCO levelling dataset was used as a reference for almost all of them. The clutter objects were identified from images by using the Mask R-CNN algorithm which was trained with the MS-COCO dataset, and the location of the clutter objects in the point cloud frame was identified in terms of the spatial parameter. By applying the method on 10 sets of the KITTI dataset, the results showed an overall accuracy of 91%, error of omission for clutter objects 7%, error of commission for clutter objects 72%, error of omission for map 22%, and error of commission for map 0% (0.448%). Previously, removing clutter objects from a point cloud was a labor-intensive task which involved the purchase of a cloud editor and subsequent manual removal. However, the proposed method allowed the researchers to skip those steps and automatically remove the clutter objects. The accuracy of the method is highly dependent on the accuracy of the image segmentation, which is expected to improve in future and consequently the method’s accuracy will also be gradually improved. Moreover, in terms of utilizing MMS data, as RGB information have enhanced the ability to segment point clouds from point cloud map in this study, the position and intensity information which can only obtained from LiDAR point cloud may improve image segmentation ability [19].

## Figures and Tables

**Table 1:** Summary of KITTI dataset used.

Name	Included Class Labels
2011_09_26_drive_0001	12 cars/ 2 cyclists/ 1 tram
2011_09_26_drive_0011	15 cars/ 1 van/ 1 truck/1 pedestrian/ 1 cyclist
2011_09_26_drive_0014	26 cars/ 4 vans/ 1 truck/ 5 pedestrian/ 4 cyclists/ 1 tram
2011_09_26_drive_0017	4 cars
2011_09_26_drive_0018	11 cars/ 2 vans/ 2 trucks
2011_09_26_drive_0048	7 cars/ 1 van
2011_09_26_drive_0051	26 cars/ 15 vans/ 1 truck/ 3 pedestrians/ 2 cyclists/ 1 tram
2011_09_26_drive_0052	4 cars/ 4 vans/ 1 truck
2011_09_26_drive__0056	13 cars/ 3 vans/ 1 truck/ 2 pedestrians/ 1 cyclist/ 6 trams
2011_09_26_drive_0059	52 cars/ 3 vans/ 5 pedestrians

**Table 2:** Error matrix for results of KITTI dataset No. 2011_09_26_drive_0056.

Number of Points		Reference Data			Error of Commission
		Clutter Objects	Map	Total	
**Classified Data**	**Clutter Objects**	160,810	440,463	601,273	73%
	**Map**	41,879	4,802,486	4,844,365	1%
	**Total**	202,689	5,242,949	5,445,638	-
**Error of Omission**		21%	8%	**Overall Accuracy**	
				95%	

**Table 3:** Equation for Accuracy Metrics and the application examples.

Accuracy Metrics	Equation	Application Example for TABLE II (%)
Overall Accuracy	$\frac{Number\;of\;correctly\;classified\;points}{Total\;number\;of\;reference\;points}$	$\frac{\left(160,810+4,802,486\right)}{5,445,638}$ =95
Error of Omission for Clutter Objects	$\frac{Number\;of\;incorrectly\;classified\;noise\;points}{Total\;number\;of\;reference\;noise\;points}$	$\frac{41,879}{202,689}=21$
Error of Commission for Clutter Objects	$\frac{Number\;of\;incorrectly\;classified\;map\;points}{Total\;number\;of\;classified\;noise\;points}$	$\frac{440,463}{601,273}=73$
Error of Omission for Map	$\frac{Number\;of\;incorrectly\;classified\;map\;points}{Total\;number\;of\;reference\;map\;points}$	$\frac{440,463}{5,242,949}=8$
Error of Commission for Map	$\frac{Number\;of\;incorrectly\;classified\;noise\;points}{Total\;number\;of\;classified\;map\;points}$	$\frac{41,879}{4,844,365}=1$

## Data Availability

The data that support the findings of this study are openly available in “KITTI dataset” at https://doi.org/10.1177/0278364913491297, reference number 2. The data that support the findings of this study are openly available in “Common Objects in Context” at http://cocodataset.org/#download, reference number 11. The code supports the findings of this study are available in github at https://github.com/matterport/Mask_RCNN, reference number 12.
